# The new pyridoindole antioxidant SMe1EC2 and its intervention in hypoxia/hypoglycemia-induced impairment of longterm potentiation in rat hippocampus

**DOI:** 10.2478/v10102-011-0011-0

**Published:** 2011-03

**Authors:** Zdenka Gáspárová, Vladimír Šnirc, Svorad Štolc

**Affiliations:** 1 Institute of Experimental Pharmacology & Toxicology, Slovak Academy of Sciences, SK-84104 Bratislava, Slovak Republic; 2 BIONT, Inc., Bratislava, Slovak Republic

**Keywords:** oxidative stress, hippocampus, long-term potentiation, pyridoindole, rat

## Abstract

Previously, the pyridoindole SMe1EC2 was proved to inhibit lipoperoxidation and carbonylation of proteins in rat brain cortex in the system Fe^2+^/ascorbate and improvement of resistance of the rat hippocampus was reported against ischemic conditions *in vitro* (hypoxia/hypoglycemia) expressed by the enhanced neuronal response recovery in reoxygenation. The hippocampus fulfils many of the criteria for a neuronal correlate of learning and memory. Recently, an impairment of hippocampal long-term potentiation (LTP) was reported under oxidative stress. Different therapies, including antioxidants, have been studied intensively concerning the impairment of neuronal plasticity. In this study marked reduction of LTP, elicited by a single burst (100 Hz, 1s) in the CA3-CA1 area of rat hippocampal slices, was shown due to transient hypoxia/hypoglycemia compared to control slices. On the basis of previously reported antioxidant and neuroprotective effects of SMe1EC2, its effect on loss of LTP in the hippocampus due to ischemic conditions was studied *in vitro*. The pyridoindole tested improved hypoxia/hypoglycemia-induced reduction of LTP compared to untreated hypoxic slices. An opposite effect of SMe1EC2 on LTP induction was found in control slices. The mechanism of SMe1EC2 action on LTP in ischemic conditions has been suggested to differ from the mechanism of its effect in “normoxia” and may be due to different redox status in control and ischemic brain tissue. The manifested LTP-protective effect of SMe1EC2 observed in the rat hippocampus exposed to ischemia *in vitro* may find exploitation in therapy associated with injured neuronal plasticity in some conditions, including ischemia, trauma and aging in man.

## Introduction

Recently it has been generally accepted that oxidative stress is one of the multiple mechanisms participating in brain damage and it has been implicated in the pathogenesis of chronic neurodegenerative disorders like Alzheimer and Parkinson diseases (Keller *et al*., 2005; Mariani *et al*., 2005; Moreira *et al*., 2006) and in acute brain injuries such as ischemia or head trauma (Sopala *et al*., 2000; Dai *et al*., 2007; Tariq *el al*., 2010). Oxidative stress is believed to be one of the aspects contributing to a decline in function of the central nervous system during acute and chronic neuronal damage and aging. Traumatic brain injury produces learning and memory deficits that are thought to be mediated partially by impaired hippocampal function. Long-term potentiation (LTP), a long-lasting increase in synaptic strength of monosynaptic pathways in the hippocampus, makes it an attractive model to study cellular mechanisms that may participate in learning and memory formation.

Neuronal tissue is equipped with protective enzymatic and non-enzymatic defensive anti-oxidative mechanisms which offer a partial resistance to oxidative stress. Disturbance of the balance between the production of reactive oxygen species and the capacity of protective antioxidants produces neuronal damage. The idea of neuroprotective therapy for acute ischemic stroke is based on results from extensive studies on animal models of brain ischemia, demonstrating efficacy of many natural and synthetic agents. Therapy using antioxidants is intensively studied. Contrary to positive conclusions with antioxidants from experimental animal models, clinical experience failed to find neuroprotectants efficient in human stroke, infarction, brain trauma, tissue preservation, etc. Novel approaches and carefully designed trials are needed to disclose more potent drugs with neuroprotective and recovery enhancing effects (Fisher, 2011).

In the Institute of Experimental Pharmacology and Toxicology, Slovak Academy of Sciences, Slovakia, the bearing issue is focused on the search for new compounds with antioxidant and antiradical properties. This research was based on stobadine, the compound revealing remarkable antioxidant, radical scavenging, and tissue protective action (Horáková and Štolc, 1998). Sites in the pyridoindole stobadine molecule responsible for antioxidant and antiradical properties were identified and new derivatives have been prepared by an appropriate substitution (Štolc *et al*., 2008); some results have been included in the Slovak patent No. 287506 (Štolc *et al*., 2010). One of the new derivatives is the compound 2-ethoxycarbonyl-8-methoxy-2,3,4,4a,5,9b-hexahydro-1*H*-pyrido-[4,3b]indolinium chloride, code SMe1EC2, substituted with methoxy-group on the aromatic cycle and ethoxycarbonyl-group substituted in position 2- of the piperidine nitrogen. The pyridoindole SMe1EC2 was found to have higher antioxidant capability than the parent drug stobadine, at simultaneous elimination of the alpha_1_-adrenolytic activity considered as undesired side effect and it displayed a markedly decreased acute toxicity compared to stobadine after *p.o., i.p.* and *i.v.* administration to mice (Table 1). Beneficial effects of this prospective pyridoindole have been established (Štolc *et al*., 2008) and they are briefly summarized in Table 2. This compound revealed remarkably higher inhibitory effect on lipoperoxidation in rat brain homogenates in the presence of Fe^2+^/ascorbate system than did stobadine. Similarly, the inhibitory effect of SMe1EC2 on oxidative impairment of creatine phosphokinase in rat brain homogenate exposed to Fe^2+^/ascorbate system was of higher potency than that of stobadine. The neuroprotective action of the pyridoindole SMe1EC2 was found also in the model of acute head trauma in mice where diminished sensomotoric impairment (expressed as “time on wire”), eliminated the increase in brain wet weight, which could be ascribed to acute brain edema. Along with this, the incidence of subdural bleeding, brain parenchyma bleeding, and bleeding into brain chambers were significantly reduced in treated mice. Administration of SMe1EC2 fully prevented a decrease in brain total glutathione level elicited by trauma. Recently SMe1EC2 was found to inhibit formation of protein carbonyl groups induced by the Fe^2+^/ascorbate system in brain cortex homogenates of rats (Gáspárová *et al*., 2010a). The improvement of neuronal function recovery in reoxygenation after transient exposure to hypoxia/hypoglycemia of rat hippocampal slices was observed in several studies regardless the way of its administration (Gáspárová *et al*., 2008; 2009; Gáspárová *et al*., Gáspárová *et al*., 2010b). The new pyridoindole has been suggested to apply its neuroprotective action in different events connected to oxidative injury. In this study, we examined its action in impairment of neuronal plasticity due to exposure to transient hypoxia/hypoglycemia in the rat hippocampus, a brain region particularly vulnerable to oxidative stress, yet also exceptionally plastic.

**Table 1:** Comparison of SMe1EC2 and stobadine.

Action	SMe1EC2	Stobadine
**Anti-lipoperoxidation effect** (rat brain homogenate)	pIC_50_=5.487±0.014	pIC_50_=4.469±0.023
**Inhibition of creatine phosphokinase oxidative impairment** (rat brain homogenate)	2.284	1 (equivalent activity)
**Alpha-adrenolytic effect** (rat aortic rings)	no effect	pA_2_=7.26±0.12
**Acute toxicity** (mouse)	LD_50_>2400 mg/kg, *p.o*.	LD_50_=323.68 mg/kg, *p.o*.
	LD_50_=1963.36 mg/kg, *i.p*.	LD_50_=164.44 mg/kg, *i.p*
	LD_50_=181.13 mg/kg, *i.v.*.	LD_50_=63.13 mg/kg, *i.v*.

**Table 2:** Neuroprotective and antioxidant action of SMe1EC2.

Model of injury	Effect of SMe1EC2	Animal/Tissue
**Fe**^**2+**^**/ascorbate**	Inhibitory effect on lipoperoxidation	rat/brain homogenate
**Fe**^**2+**^**/ascorbate**	Inhibitory effect on oxidation of creatine phosphokinase	rat/brain homogenate
**Fe**^**2+**^**/ascorbate**	Inhibitory effect on formation of protein carbonyl groups	rat/brain cortex homogenate
**Acute head trauma**	Improvement of sensomotoric stage	mouse
**Acute head trauma**	Reduction in brain edema	mouse/brain
**Acute head trauma**	Reduction of bleeding into brain	mouse/brain
**Acute head trauma**	Prevention of injury-induced decrease in total glutathione level	mouse/brain homogenate
**Acute head trauma**	Elimination of injury-induced increase in total lactate level	mouse/brain homogenates
**Hypoxia/hypoglycemia**	Improved recovery of neuronal response in reoxygenation	rat/hippocampal slices
**Hypoxia/hypoglycemia**	Reduction of edema	rat/hippocampal slices

## Methods

### Animals

Male Wistar rats, 2 months old, weight 221±11 g, n=40, from the breeding station Dobrá Voda (Slovak Republic, reg. No. SK CH 4004) were used. The rats had free access to water and food pellets and were kept on 12/12 h light/dark cycle. Animals were acclimated one week prior to the experiments. All procedures involving animals were performed in compliance with the Principles of Laboratory Animal Care issued by the Ethical Committee of the Institute of Experimental Pharmacology and Toxicology, Slovak Academy of Sciences and by the State Veterinary and Food Administration of Slovakia.

### Drug

The pyridoindole derivative SMe1EC2 was synthetized in the Institute of Experimental Pharmacology and Toxicology, Slovak Academy of Sciences, Slovakia.

### Rat hippocampal slices preparation and LTP induction

The rats were briefly anesthetized by ether, decapitated and the hippocampus was quickly removed from the brain and cut into 400 µm thick slices with the McIllwain Tissue Chopper. The slices were kept in the holding chamber for at least 1-h recovery period before the experiment started. During the experiments, the slice was kept in the recording chamber and continuously perfused with artificial cerebrospinal fluid (ACSF) bubbled with 95% O_2_ and 5% CO_2_ at the constant rate monitored by aquatic manometer. Oxygen/glucose deprivation was obtained by replacing the gas mixture with O_2_ by the gas mixture with N_2_ by switching the valves, along with superfusion of the slices with ACSF equilibrated with the oxygen-free gas mixture and diminished glucose from 10 to 4×10^−3^ mol/l. The temperature of the recording chamber was kept at 35.0–35.5°C. Neurons were stimulated via Schaffer collaterals in the CA3 region and field excitatory postsynaptic potentials (fEPSPs) were recorded extracellularly from the pyramidal cell layer in the CA1 region. To measure baseline synaptic transmission, the stimuli were applied every 20 s. The stimulus intensity was reduced to 50% or less of the fEPSP amplitude when a population spike generation started to be detected. The slices were stabilized for about 15 min, the drug tested was applied during 30 min before high-frequency stimulation (HFS) in “normoxic” slices, or 30 min before transient hypoxia/hypoglycemia in hypoxic slices, and then during the whole experiment. Hypoxic slices were exposed to short 3.5 min hypoxia/hypoglycemia followed by 20 min reoxygenation. LTP was induced by a single 100-Hz train with 1000ms train duration. After HFS, the baseline stimulation recording continued for at least 60 min.

### Statistical analysis

The electrophysiological measurements were done from 6–10 rats in each experimental group. Three stimuli per minute were averaged in off-line analysis. The normalized value 1 represents the mean fEPSP amplitude recorded during a 10-min period before HFS in each slice. In the figures, each value represents the mean fEPSP amplitude (± SEM) at a given time from 6–13 hippocampal slices. The mean values for the last 10 min (50–60 min after HFS) for each group were calculated and compared. Statistical significance of differences between these values was established by Student′s t-test and *p-*values below 0.05 were considered statistically significant.

## Results

### Effect of SMe1EC2 and LTP-induction in control and hypoxic rat hippocampal slices

**Figure 1:**
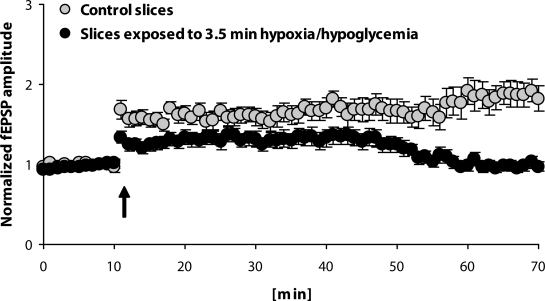
LTP induced by a single burst (100 Hz, 1s) in CA3-CA1 pathway of control rat hippocampal slices (n=13) compared to the same response in slices (n=10) exposed to 3.5-min hypoxia/hypoglycemia and after 20 min to high-frequency stimulation. Arrow shows the application of HFS. Values express mean±SEM.

LTP was elicited by a single burst (100 Hz, 1 s) with the magnitude 186.0±15.8% measured within the last 50–60 min after HFS in the control (“normoxic”) rat hippocampal slices, compared to the mean baseline fEPSP amplitude measured during 10 min before HFS. In the slices exposed to 3.5-min hypoxia/hypoglycemia, transient impairment or complete cessation of synaptic transmission during hypoxia was observed with almost complete recovery in 20-min reoxygenation (not shown). This short exposure of slices to hypoxia/hypoglycemia resulted in marked impairment of LTP (Figure 1). Immediately after HFS, the increase of fEPSP response due to HFS was smaller compared to that in control slices, as well as to that during further 60 min of measurement. The mean fEPSP amplitude within 50–60 min after HFS was similar to that before HFS in hypoxic slices.

**Figure 2:**
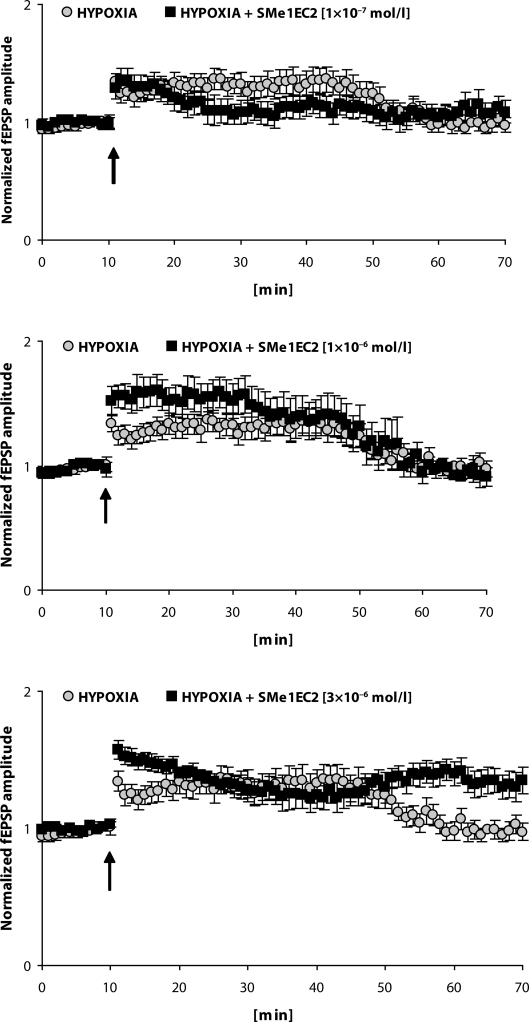
LTP induced by a single burst (100 Hz, 1s) in CA3-CA1 pathway of slices (n=10) exposed to 3.5-min hypoxia/hypoglycemia and after 20 min to high-frequency stimulation compared with LTP in hypoxic slices treated 30 min before hypoxia/hypoglycemia and during whole experiment by the pyridoindole SMe1EC2 (1×10^−7^; 1×10^−6^; 3×10^−6^ mol/l; n=9; n=8; n=8, respectively). Arrow shows the application of HFS. Values represent mean±SEM.

The effect of SMe1EC2 on LTP induction in hypoxic slices is shown in Figure 2. In the concentration of 3×10^−6^ mol/l a significant improvement in induction of LTP was observed in hypoxic slices treated by SMe1EC2 compared to the response in hypoxic untreated slices. Both low concentrations of SMe1EC2 tested failed to affect the impairment of LTP in the hippocampus after experimental ischemia *in vitro*.

**Figure 3:**
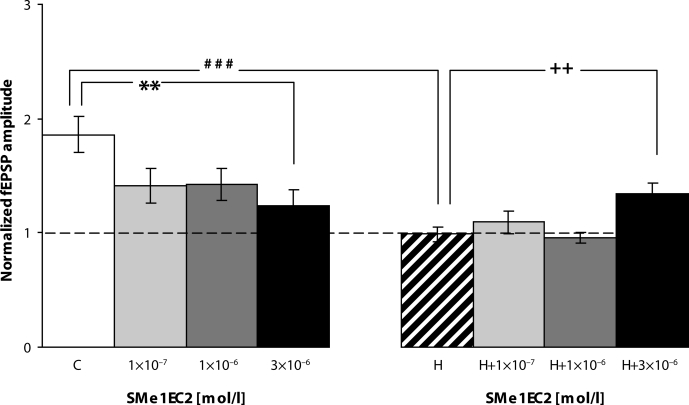
Normalized fEPSP amplitude measured in 50–60 min after HFS in control slices (C), control slices treated with SMe1EC2, hypoxic untreated slices (H) and hypoxic slices treated with SMe1EC1 in concentration of 1×10^−7^; 1×10^−6^ and 3×10^−6^ mol/l. Significant difference between fEPSP amplitude in control slices compared to hypoxic slices (^###^*p=*0.0001), between controls and control slices treated with SMe1EC2 (3×10^−6^ mol/l) (**p=0.0187), and between hypoxic slices compared to hypoxic slices treated with SMe1EC2 (3×10^−6^ mol/l) (^++^ *p=*0.0058). Significant difference was calculated by Student t-test.

The compound SMe1EC2 (1×10^−7^; 1×10^−6^ mol/l), present 30 min before HFS, appears to reduce LTP magnitude in “normoxic” slices 50–60 min after HFS and this reduction was significant in the highest concentration tested (3×10^−6^ mol/l, *p=*0.0187). The mean fEPSP amplitudes measured during 50–60 min after HFS are summarized in Figure 3.

## Discussion

Loss of LTP in the hippocampus was observed in traumatic brain injury (Miyazaki *et al*., 1992; Reeves *et al*., 1995; Sick *et al*., 1998; Sanders *et al*., 2000; Schwarzbach *et al*., 2006), after experimental subarachnoid hemorrhage (Tariq *et al*., 2010), after ischemia (Ito *et al*., 1994; Sopala *et al*., 2000; Dai *et al*., 2007) and after hypoxia/hypoglycemia (Gáspárová *et al*., 2008). In human CNS tissue and rodent brain similar molecular mechanisms of LTP were reported (Cooke & Bliss, 2006).

The results presented in this article show that transient 3.5-min hypoxia/hypoglycemia of rat hippocampal slices resulted in significant reduction of LTP. These results are in good agreement with the observation that extracellular acidosis, which is associated with several pathological conditions in the CNS (like ischemia, hypoxia and neuronal injury), suppresses LTP induction (Velísek, 1998). An impairment of hippocampal LTP was also found in model oxidative stress induced by hydrogen peroxide in rats (Maalouf & Rho, 2008).

Neuroprotection from ischemic brain damage by diets was examined in animal models and in humans. Nutritional intervention due to antioxidants and polyphenolic compounds from fruits, vegetables, nuts and grains decreases markers of oxidative damage, such as malondialdehyde and protein carbonyls, and decreases levels of proinflammatory cytokines either directly or indirectly by reducing oxidative damage (Gemma *et al*., 2007). Vitamin E has been shown to improve age-related impairment in LTP (Murray & Lynch, 1998), its administration improved the learning and memory ability of mouse offspring whose mothers were exposed to tabacco smoke during pregnancy (Yang *et al*., 2008) and improved cognitive behaviors (Joseph *et al*., 1999; Joseph *et al*., 1998). The effect of other antioxidants on LTP-impairment was tested in several studies. Oxidative impairment of hippocampal LTP induced by H_2_O_2_ was prevented by ketone bodies (Maalouf & Rho, 2008), which exert antioxidant effect in experimental model of neurological diseases. Similarly, the compound U-92032U-92032a Ca^2+^ channel blocker and antioxidant, preserved LTP in hippocampal CA1 neurons when administered to gerbils 1 h prior to bilateral carotid artery occlusion (Ito *et al*., 1994).

Thus there are several implications about putative neuroprotective action of antioxidants in loss of LTP due to oxidative stress. We focused on the effect of the compound SMe1EC2, the new pyridoindole derivative with antioxidant properties. Previously, the neuroprotective action of SMe1EC2 expressed by improved recovery of neuronal transmission in reoxygenation was found in rat hippocampal slices exposed to model ischemic conditions *in vitro*. Such an improvement of functional recovery was observed after transient hypoxia/hypoglycemia in different experiments where SMe1EC2 was: (1) applied into superfusing medium (3×10^−8^–3×10^−6^ mol/l); (2) after 10-day oral treatment of adult rats (50 and 250 mg/kg/day, *p.o*.), and (3) in offspring after 18-day treatment of the mothers (50 and 250 mg/kg/day, *p.o*.) (Gáspárová *et al*., 2008; 2009; 2010b). The present data showed improved LTP due to SMe1EC2 treatment in hypoxic rat hippocampal slices and reduction of LTP in “normoxic” slices. Similar results were found with melatonin, which impaired LTP in the dentate gyrus area of the hippocampus and induced learning and memory deficit in control rats (Cao *et al*., 2009). Accordingly, administration of alpha-lipoic acid to control animals resulted in a significant impairment of LTP amplitude (Wang *et al*., 2008). The differential effect of antioxidants on LTP in control and hypoxic hippocampal slices may be due to the different redox status in control and ischemic brain tissue. The neuroprotective effect of SMe1EC2 during hypoxia/hypoglycemia may be explained by its high anti-lipoperoxidation activity, which presumably contributes to the preservation of the neuronal cell membrane and to its permeability, and further by its inhibitory effect on carbonylation of proteins mediated by conditions involving oxidative stress, and thus SMe1EC2 might protect neurons from damage of membrane lipids and protein receptors. The beneficial effect of SMe1EC2 on improved LTP in treated hypoxic hippocampal slices probably does not act by direct interaction with glutamate receptor. This assumption is based on results with its parent drug stobadine, which did not compete [^3^H]glutamate binding in rat brain membranes (Kvaltínová & Štolc 1997; Gáspárová-Kvaltínová & Štolc, 2003).

## Conclusion

We conclude that the LTP-protective effect of SMe1EC2 found in the rat hippocampus exposed to model ischemia may prove beneficial in therapeutic application when neuronal plasticity is injured in some conditions including ischemia, trauma and aging in man. The mechanism of pyridoindole antioxidant effect in ischemic conditions may differ from the mechanism of its effect in control “normoxic” conditions.
